# Development of Machine Learning–Based Risk Prediction Models to Predict Rapid Weight Gain in Infants: Analysis of Seven Cohorts

**DOI:** 10.2196/69220

**Published:** 2025-06-18

**Authors:** Miaobing Zheng, Yuxin Zhang, Rachel A Laws, Peter Vuillermin, Jodie Dodd, Li Ming Wen, Louise A Baur, Rachael Taylor, Rebecca Byrne, Anne-Louise Ponsonby, Kylie D Hesketh

**Affiliations:** 1 Institute for Physical Activity and Nutrition, School of Exercise and Nutrition Sciences, Deakin University, Geelong, Australia; 2 School of Health Sciences, Faculty of Health & Medicine, UNSW Sydney, Wallace Wurth Building, Kensington, 2330, Australia, 61 0290659337; 3 Barwon Health, Geelong, Australia; 4 Discipline of Obstetrics and Gynaecology, The Robinson Research Institute, The University of Adelaide, Adelaide, Australia; 5 School of Public Health and Sydney Medical School, The University of Sydney, Sydney, Australia; 6 Department of Medicine, University of Otago, Dunedin, New Zealand; 7 School of Exercise and Nutrition Sciences, Faculty of Health, Queensland University of Technology, Kelvin Grove, Australia; 8 The Florey Institute of Neuroscience and Mental Health, Murdoch Children's Research Institute, Royal Children's Hospital, The University of Melbourne, Parkville, Australia

**Keywords:** infants, rapid weight gain, risk prediction, machine learning, childhood obesity, pooled analysis

## Abstract

**Background:**

Rapid weight gain (RWG) during infancy, defined as an upward crossing of one centile line on a weight growth chart, is highly predictive of subsequent obesity risk. Identification of infant RWG could facilitate obesity risk assessment from infancy.

**Objective:**

Leveraging machine learning (ML) algorithms, this study aimed to develop and validate risk prediction models to identify infant RWG by the age of 1 year.

**Methods:**

Data from 7 Australian and New Zealand cohorts were pooled for risk model development and validation (n=5233). A total of 8 ML algorithms predicted infant RWG using routinely available prenatal and early postnatal factors, including maternal prepregnancy weight status, maternal smoking during pregnancy, gestational age, parity, infant sex, birth weight, any breastfeeding and timing of solids introduction at the age of 6 months. Pooled data were randomly split into a training dataset (70%) and a test dataset (30%) for model training and validation, respectively. Model consistency was evaluated using 5-fold cross-validation. Model predictive performance was evaluated by area under the receiver operating characteristic (ROC) curve (AUC), accuracy, precision, sensitivity, specificity, and Cohen κ.

**Results:**

The average prevalence of infant RWG was 27%. In the training dataset, all ML algorithms showed acceptable to excellent discrimination with AUCs ranging from 0.75 to 0.86. Accuracy, which indicates the overall correctness of the model, ranged from 0.69 to 0.78. Precision, which measures the model’s ability to avoid false positives, ranged from 0.68 to 0.77. The spread of sensitivity, specificity, and Cohen κ of all models was 0.68‐0.80, 0.65‐0.78, and 0.38‐0.56, respectively. Of the 8 algorithms, the Gradient Boosting model showed the most favorable predictive accuracy. Validation of the Gradient Boosting model in the testing dataset exhibited excellent discrimination (AUC 0.3‐0.6) and good ability to make accurate predictions, particularly true positive cases (with accuracy and sensitivity>0.75), but modest performance for precision (0.57‐0.60) and Cohen κ (0.47‐0.52).

**Conclusions:**

This study developed the first set of ML-based risk prediction models to identify infants’ risk of experiencing RWG by the age of 1 year with acceptable accuracy. The models could be feasibly integrated into routine child growth monitoring and may facilitate population-wide early obesity risk assessment in primary health care.

## Introduction

Overweight and obesity are an intractable global challenge with substantial health consequences [1]. Compelling evidence suggests that obesity risk originates early in life and is hard to reverse once developed [2]. World Health Organization (WHO) global estimates indicate that a high prevalence of overweight and obesity is already evident in children younger than 5 years, with 37 million cases being reported in 2022 [3]. The WHO has thus recognized “Lowering the prevalence of overweight in children under the age of five” as one of the key outcome targets in the acceleration plan to stop obesity [4]. Identification of obesity risk from early life along with timely delivery of tailored interventions, are therefore strongly recommended to achieve effective and sustained impact.

Extensive research has attempted to develop obesity risk prediction models to identify high-risk populations [5], mostly in older children and adults when obesity and modifiable risk factors are already established [6,7]. Despite some models having been developed to predict early childhood obesity risk, these models are largely derived from a single cohort using statistical approaches and are often constrained to linear relationship assumptions, resulting in suboptimal model prediction accuracy and low generalisability [8]. A key consideration for developing clinically useful risk prediction models is clinical impact and ease of clinical integration [9]. It is not possible to evaluate the clinical impact of these models due to the costs and ethical reasons associated with long-term follow-up and allowing children to develop obesity without intervention. Moreover, widespread obesity stigma and the reluctance to label children as having obesity further impede the clinical integration of existing obesity risk models [10,11]. Thus, novel approaches to facilitate early obesity risk identification, communication, and model integration in clinical practice are needed.

Convincing evidence suggests that accelerated physical growth in early life is highly predictive of later obesity and cardiometabolic outcomes [12,13]. Several systematic reviews concluded that rapid weight gain (RWG) from birth to the age of 1 year, defined as the upward centile crossing in a weight growth chart, was associated with a fourfold higher risk of later obesity than those without infant RWG [14,15]. As such, infant RWG is considered a sensitive proxy marker that denotes future obesity risk. A risk prediction model to predict infant RWG could facilitate early obesity risk identification from infancy and the timely delivery of interventions before adverse health behaviors are established. Furthermore, such a model could be easily evaluated and integrated in primary health care where infant growth is already routinely monitored. To improve risk prediction accuracy, we propose the use of machine learning (ML), a type of artificial intelligence technique that is powerful at making accurate predictions. Reviews have indicated that ML models outperform statistical models for obesity risk prediction [16,17]. Using data from 7 Australian and New Zealand cohorts, we used ML to develop and validate risk prediction models to identify infants at risk of experiencing RWG by the age of 1 year.

## Methods

### Data Source and Variables

Data from 7 Australian and New Zealand cohorts: Barwon Infant Study (BIS) [18], Healthy Beginnings [19,20], Infant Feeding Activity and Nutrition Trial (InFANT) [21,22], InFANT Extend [23], LIMIT [24,25], NOURISH [26,27], and Prevention of Overweight in Infancy (POI) were used [28,29]. Baseline recruitment of all studies was undertaken either antenatally (15‐34 wk gestation) or postnatally (between ages 3‐4 m) from 2007 to 2012. The 7 studies include 6423 parent-infant dyads from diverse geographical areas at the study baseline. BIS is a prospective birth cohort. The remaining studies are intervention studies targeting nutrition and physical activity in infants or pregnant women. Detailed study characteristics of the cohorts were published elsewhere [30,31]. None of the intervention studies showed differences in infant growth outcomes between intervention and control groups by the age of 1 year [32], and thus groups were combined to study prognosis [33].

### Rapid Weight Gain During Infancy

All cohorts collected child anthropometrics at birth and around 1 year of age. Child length and weight at birth were transcribed from child health records. Child length and weight around the age of 1 year were measured by trained staff. WHO growth standards were used to calculate age- and sex-specific weight-for-age *z* scores at birth and around the age of 1 year, and the difference between the two time points was calculated. RWG during infancy was defined as a change in weight-for-age *z* score>0.67, which is clinically equivalent to upward crossing one centile line in a weight growth chart [14,15].

### Selection of Predictor Variables

A range of prenatal and early postnatal factors were collected across all the cohorts. Prenatal factors include maternal country of birth (native-born vs overseas-born), an education level (tertiary vs nontertiary), smoking during pregnancy (smoker vs nonsmoker), and pre or early-pregnancy BMI (kg/m^2^). Child early postnatal factors included sex (boys vs girls), birth weight (kg), gestational age (weeks), parity (≥1 sibling vs no sibling), any breastfeeding (yes vs no) at age 6 months, and whether introduced solids (yes vs no) at age 6 months.

The selection of predictor variables for risk prediction model development was informed by previous findings on determinants of infant RWG, the availability of data or ease of data collection at primary health care visits, and the level of data missingness. Our previous statistical findings in the 7 cohorts assessed maternal prepregnancy BMI, smoking during pregnancy, country of birth, gestational age, parity, infant sex, birth weight, breastfeeding, and solids introduction at age 6 months as potential predictors of RWG by the age of 1 year [30], and these were also considered as potential predictors for the current risk prediction model development. Continuous variables capture more nuanced information compared with categorical variables, hence, continuous forms of maternal prepregnancy BMI, gestational age, and birth weight were used for risk prediction whenever possible. Maternal country of birth was not included as a predictor as it had missing data >40%, and missing data imputation would not be reliable. A total of 3 models were purposely chosen. Model 1 is limited to variables that are collected as part of the antenatal and child health records in Australia or can be easily collected at the primary health care visits: maternal prepregnancy BMI, smoking during pregnancy, gestational age, parity, infant sex, and birth weight. Infant feeding at age 6 months is not routinely collected in Australia. However, given the vital contribution of infant feeding, especially breastfeeding, in determining the risk of infant RWG, we conducted 2 additional models to further incorporate the 2 infant feeding variables. Model 2 included model 1 variables plus any breastfeeding at the age of 6 months. Model 3 additionally included solids introduction at age 6 months, upon model 2. The 3 models also enable the comparison of risk prediction accuracy at birth versus at 6 months postnatal.

### Model Development and Validation

Detailed model development and validation processes are summarised in Supplementary Figure 1 in Multimedia Appendix 1. All ML models were trained and validated using Python 3.11.8 and Scikit Learning Tool Kit library 1.6.1. Data from 7 cohorts were pooled and randomly split into a training dataset (70%) and a testing dataset (30%) [34]. The training dataset was used for model development and internal validation, from which the best-performing model was selected. The testing dataset was reserved for external validation to assess how the best-performing model performed with the new data. Missing data imputation was conducted to impute predictors with missing data: maternal prepregnancy BMI, smoking, gestational age, breastfeeding, and solids introduction. Maternal smoking during pregnancy (yes vs no) was imputed by mode imputation [35], whereby missing values were replaced with the most frequent category observed in the dataset. Median imputation was used to impute missing data on maternal prepregnancy BMI and gestational age. For breastfeeding and solids introduction, we adopted ML algorithms to impute missing values using infant sex, birth weight, and parity as predictors and those with complete data as the training dataset for missing data prediction. Detailed missing data imputation methods are provided in Supplementary Table 1 in Multimedia Appendix 1. All predictor variables were standardized before risk prediction, ensuring all variables were in the same scale to facilitate convergence speed and interpretability.

For model development in the training dataset, 8 ML algorithms widely used in study prognosis in health research were explored: Logistic Regression, Decision Tree, Random Forest, AdaBoost, Gradient Boosting, K-Neighbours, Support Vector, and Multi-Layer Perceptron [16]. The hyperparameter (configuration setting) of each model were optimized using the grid search cross-validation technique, which systematically finds the best combination of hyperparameter for a ML model [36]. The grid search cross-validation technique reduces overfitting and provides more reliable hyperparameter selection than manual tuning. Detailed model hyperparameters are presented in Supplementary Table 2 in Multimedia Appendix 1. For model training and internal validation, 5-fold cross-validation was conducted, where the training dataset was randomly split into 5 blocks of equal size (fold), and 5 independent iterations of training and internal validation were conducted, so every block of data was used for both training and internal validation [37]. Imbalanced outcome data (ie, higher proportion of infants without RWG and vs with RWG) can result in biased risk prediction models with better performance in predicting infants with no RWG. We used the Synthetic Minority Over-sampling Technique (SMOTE) [38], a commonly used method in ML, to address imbalanced outcome data. SMOTE generates synthetic samples of infants with RWG that are combinations of existing RWG infants, thereby balancing the outcome distribution and improving the prediction of infants with RWG. SMOTE was exclusively performed on the training folds, which prevents data leakage, eliminates the risk of overestimating the model’s performance, and maintains a proper evaluation of the model’s generalisability (Supplementary Figure 2 in Multimedia Appendix 1). The performance metrics of five iterations were averaged to obtain the mean performance metrics of each ML model. The average model performance of 8 ML models was compared, from which the best-performing models were selected for subsequent external validation in the testing dataset.

The predictive performance of the models against the observed prevalence of infant RWG in both internal and external validation was assessed by various metrics, including receiver operating characteristic (ROC) plot, precision-recall curve (PRC) plot, accuracy, precision, sensitivity, specificity, *F*_1_-score, and Cohen κ. A ROC curve plots false positive rate (1-specificity) as the x-axis against true positive rate (sensitivity) as the y-axis at various thresholds (defined as the cutoff value at which the model determines a positive or negative prediction). The area under the ROC curve (AUC) summarizes the model’s overall ability to discriminate between infants with or without RWG across various thresholds. An AUC of 0.70‐0.79 and ≥0.80 is considered acceptable and excellent discrimination, respectively [39]. Accuracy evaluates the overall correctness of the model (both true positives and negatives). Precision examines the model’s ability to predict true positives while minimizing false positives. Sensitivity (recall) assesses the model’s ability to correctly classify true positives. The PRC plots recall (y-axis) against precision (x-axis) over different thresholds, which is particularly useful for a classification model with imbalanced outcome data. The closer the curve toward the top right corner (high precision and high recall) or the larger area under the PRC (AUPRC), the better model performance with >0.7 is generally considered as decent model performance [40]. Likewise, *F*_1_-score evaluates the balanced performance of the model that accounts for both precision and sensitivity, which is particularly useful for risk prediction with the outcome that has an uneven distribution. Specificity measures the model’s ability to correctly identify true negatives. Higher values of these metrics indicate better model performance. Cohen κ assesses agreement between predicted and observed outcomes, accounting for agreement by chance, and is also useful for imbalanced outcome data (0.61‐1.0: perfect agreement; 0.41‐0.60 moderate agreement; 0.21‐0.40 fair agreement; ≤0.20 slight or poor agreement). Detailed interpretation and calculation of these metrics are provided in Supplementary Table 3 in Multimedia Appendix 1. To enhance model transparency and interpretability, additional Shapley Additive Explanations (SHAP) analyses were performed to quantify the contribution of each predictor in the risk prediction of infant RWG.

### Ethical Considerations

Each study received ethical approval from relevant institutional ethics committees and informed written consent was obtained from participants. The original consent covers secondary analysis without additional consent. The current analysis uses deidentified data and received an ethics exemption from the Deakin University Human Research Ethics Committee (2023-033).

## Results

Of 6423 parent-infant dyads from the 7 cohorts at baseline, 81% (n=5233) with all required data were included in analyses. The mean prepregnancy BMI was 27.7 kg/m^2^. The majority of women did not smoke during pregnancy (89.1%). The mean gestational age and infant birth weight were 39 weeks and 3.5 kg, respectively. The pooled sample included an even proportion of males and females with 64.1% being first-born child. Most infants were breastfed beyond age 6 months (59.5%) and started solids at or after age 6 months (79.9%). Across the 7 cohorts, the mean age (SD) at RWG assessment ranged from 7.0 (1.9) to 13.1 (1) months [30]. The average prevalence of RWG across 7 cohorts spanned from 21.6% to 41.3% [30]. The mean age and prevalence of RWG of 7 cohorts were 10.3 (SD 3) months and 27.4%, respectively (Table 1).

**Table 1.** Characteristics of the pooled sample from 7 cohorts (N=5233).

Maternal and infant characteristics	Values
Maternal prepregnancy BMI (kg/m^2^), n; mean (SD)	5065; 27.7 (6.5)
Maternal smoking during pregnancy	
Smoker, n (%)	466 (10.9)
Nonsmoker, n (%)	3818 (89.1)
Gestational age (weeks), n; mean (SD)	4185; 39.3 (1.8)
Infant sex	
Males, n (%)	2649 (50.6)
Females, n (%)	2584 (49.4)
Infant birth weight (kg), n; mean (SD)	5233; 3.5 (0.5)
Parity, n (%)	
No sibling	3352 (64.1)
≥1 sibling	1877 (35.9)
Breastfeeding duration, n (%)	
<6 months	1975 (40.5)
≥6 months	2904 (59.5)
Timing of solids introduction, n (%)	
Before 6 months	695 (20.1)
At or after 6 months	2615 (79.9)
Rapid weight gain around the age of 1 year, n (%)	
Yes	1433 (27.4)
No	3800 (72.6)

### Internal Validation

**Figure 1.**
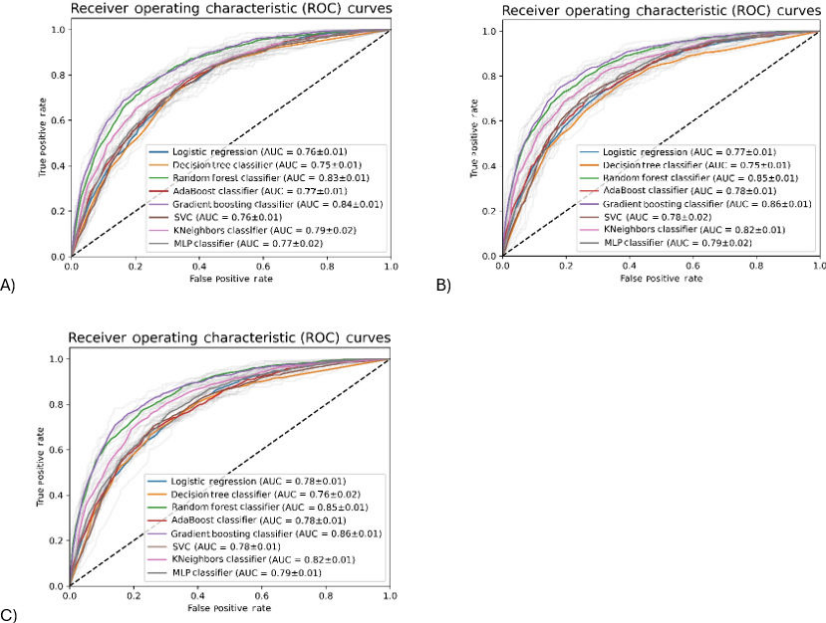
Receiver operating characteristics (ROC) curves of eight machine learning algorithms in predicting infants at risk of rapid weight gain by the age of 1 year in the training dataset (internal validation). (**A**) Model 1 included maternal prepregnancy BMI, smoking during pregnancy, gestational age, parity, infant sex, and birth weight as predictors. (**B**) Model 2 included model 1 predictors plus any breastfeeding (yes or no) at age 6 months. (**C**) Model 3 included model 2 predictors plus solids introduction (yes or no) at age 6 months. AUC: area under the receiver operating characteristic curve; MLP: multilayer perceptron; SVC: support vector classifier.

**Figure 2.**
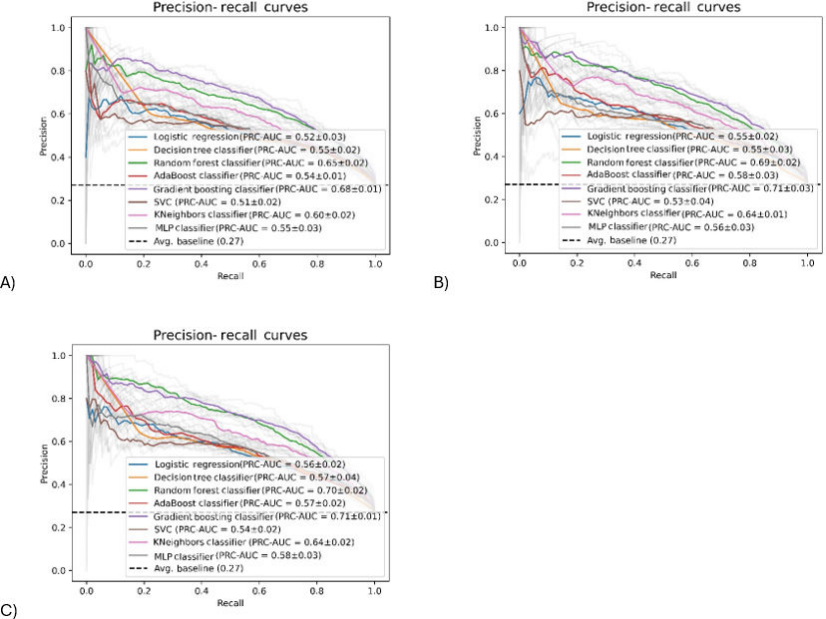
Precision recall curves (PRC) of 8 machine learning algorithms in predicting infants at risk of rapid weight gain by the age of 1 year in the training dataset (internal validation). **(A**) Model 1 included maternal prepregnancy BMI, smoking during pregnancy, gestational age, parity, infant sex, and birth weight as predictors. (**B**) Model 2 included model 1 predictors plus any breastfeeding (yes or no) at age 6 months. (**C**) Model 3 included model 2 predictors plus solids introduction (yes or no) at age 6 months. AUC: area under the receiver operating characteristic curve; MLP: multilayer perceptron; SVC: support vector classifier.

Visual inspection showed that ROC curves for all models (Figure 1) deviated from the diagonal line and were arched toward the top-left corner of the plot, indicating good discriminative ability. All 3 models showed similar AUCs with values ranging from 0.76‐0.85 (model 1), 0.75‐0.86 (model 2), and 0.76‐0.86 (model 3). Among the 8 ML algorithms, the Gradient Boosting classifier showed the best performance in terms of AUC (0.84‐0.86) in all models, suggesting an “excellent” ability to distinguish between those with and without RWG. Other predictive performance metrics of the models are shown in Table 2. The accuracy of models 1, 2, and 3 ranged from 0.69‐0.76, 0.70‐0.77, and 0.70‐0.78, respectively, which indicates that the models correctly predicted the outcomes for 69%‐78% of infants. Likewise, the 3 sets of models demonstrated similar precision of 0.68‐0.75 (model 1), 0.68‐0.76 (model 2), and 0.67‐0.77 (model 3), indicating that the models correctly predicted 67%‐77% of positive cases while showing good ability to minimize false positives. In addition, models showed good ability to identify infants with RWG as indicated by sensitivity of 0.68‐0.79 (model 1), 0.69‐0.80 (model 2), 0.69‐0.80 (model 3). The AUC range of PRC for models 1 to 3 was 0.51‐0.68, 0.53‐0.71, and 0.54‐0.71, respectively (Figure 2). For *F*_1_-score, the respective range for all models was 0.68‐0.76 (model 1), 0.69‐0.77 (model 2), and 0.69‐0.78 (model 3), indicating that the models exhibited a good balance between precision and sensitivity and suggesting good overall model performance with low prediction errors. Models 1, 2, and 3 had a specificity of 0.65‐0.75, 0.67‐0.76, and 0.65‐0.78, respectively, indicating a good ability to identify infants with no RWG. The range of Cohen κ for all models was 0.38‐0.53 (model 1), 0.39‐0.55 (model 2), and 0.40‐0.56 (model 3). The addition of infant feeding variables at age 6 months in models 2 and 3 showed 0.01‐0.03 higher increase across all predictive performance metrics when compared with model 1. Consistent with AUC results, the Gradient Boosting classifier showed the best performance in terms of accuracy, precision, sensitivity, AUPRC, *F*_1_-score, specificity, and Cohen κ.

**Table 2.** Predictive performance metrics of 8 machine learning algorithms in predicting infants at risk of rapid weight gain by the age of 1 year in the training dataset (internal validation).

Machine learning algorithms	Accuracy	Precision	Sensitivity	*F*_1_-score	Specificity	Cohen κ
Model 1^a^, mean (SD)^b^						
Logistic regression	0.69 (0.01)	0.69 (0.03)	0.68 (0.04)	0.68 (0.01)	0.70 (0.03)	0.38 (0.02)
Decision tree classifier	0.70 (0.01)	0.68 (0.02)	0.75 (0.03)	0.71 (0.01)	0.65 (0.02)	0.40 (0.01)
Random forest classifier	0.76 (0.01)	0.74 (0.02)	0.77 (0.03)	0.76 (0.02)	0.74 (0.01)	0.51 (0.02)
AdaBoost classifier	0.69 (0.01)	0.67 (0.03)	0.73 (0.03)	0.70 (0.01)	0.65 (0.04)	0.38 (0.02)
Gradient boosting classifier	0.76 (0.01)	0.75 (0.02)	0.78 (0.03)	0.77 (0.02)	0.75 (0.02)	0.53 (0.03)
Support vector classifier	0.70 (0.01)	0.68 (0.02)	0.74 (0.02)	0.71 (0.01)	0.67 (0.02)	0.41 (0.02)
K-neighbors classifier	0.73 (0.01)	0.70 (0.02)	0.79 (0.03)	0.74 (0.02)	0.68 (0.02)	0.47 (0.02)
Multilayer perception classifier	0.71 (0.01)	0.69 (0.02)	0.74 (0.03)	0.71 (0.01)	0.68 (0.03)	0.42 (0.02)
Model 2^c^, mean (SD)						
Logistic regression	0.70 (0.01)	0.70 (0.02)	0.69 (0.02)	0.69 (0.01)	0.71 (0.02)	0.40 (0.01)
Decision tree classifier	0.70 (0.01)	0.68 (0.02)	0.72 (0.02)	0.70 (0.01)	0.67 (0.02)	0.39 (0.02)
Random forest classifier	0.76 (0.01)	0.75 (0.02)	0.77 (0.03)	0.76 (0.01)	0.76 (0.02)	0.52 (0.02)
AdaBoost classifier	0.71 (0.01)	0.69 (0.03)	0.73 (0.03)	0.71 (0.01)	0.68 (0.04)	0.41 (0.02)
Gradient boosting classifier	0.77 (0.01)	0.77 (0.02)	0.78 (0.02)	0.77 (0.01)	0.77 (0.02)	0.55 (0.01)
Support vector classifier	0.72 (0.01)	0.70 (0.03)	0.75 (0.02)	0.72 (0.02)	0.69 (0.02)	0.44 (0.03)
K-neighbors classifier	0.75 (0.01)	0.72 (0.02)	0.80 (0.03)	0.76 (0.01)	0.70 (0.02)	0.50 (0.02)
Multilayer perception classifier	0.73 (0.01)	0.71 (0.02)	0.74 (0.02)	0.73 (0.02)	0.71 (0.01)	0.46 (0.02)
Model 3^d^, mean (SD)						
Logistic regression	0.70 (0.01)	0.70 (0.02)	0.69 (0.02)	0.69 (0.01)	0.71 (0.02)	0.40 (0.01)
Decision tree classifier	0.70 (0.01)	0.67 (0.02)	0.74 (0.02)	0.71 (0.01)	0.65 (0.01)	0.40 (0.01)
Random forest classifier	0.77 (0.00)	0.76 (0.02)	0.77 (0.02)	0.76 (0.01)	0.76 (0.02)	0.53 (0.01)
AdaBoost classifier	0.71 (0.01)	0.69 (0.03)	0.74 (0.02)	0.71 (0.01)	0.68 (0.04)	0.41 (0.02)
Gradient boosting classifier	0.78 (0.00)	0.77 (0.02)	0.79 (0.01)	0.78 (0.01)	0.77 (0.01)	0.56 (0.01)
Support vector classifier	0.71 (0.01)	0.69 (0.02)	0.75 (0.02)	0.72 (0.02)	0.68 (0.02)	0.43 (0.03)
K-neighbors classifier	0.74 (0.01)	0.71 (0.02)	0.80 (0.03)	0.75 (0.01)	0.69 (0.02)	0.49 (0.02)
Multilayer perception classifier	0.73 (0.01)	0.72 (0.02)	0.73 (0.02)	0.72 (0.01)	0.72 (0.02)	0.45 (0.02)

a Model 1 included maternal prepregnancy BMI, smoking during pregnancy, gestational age, parity, infant sex, and birth weight as predictors.

b Values are presented as mean (SD) and are calculated as the average performance metrics of five iterations from the 5-fold cross validation

c Model 2 included model 1 predictors plus breastfeeding (yes or no) at age 6 months.

d Model 3 included model 2 predictors plus solids introduction (yes or no) at age 6 months.

### External Validation

**Figure 3.**
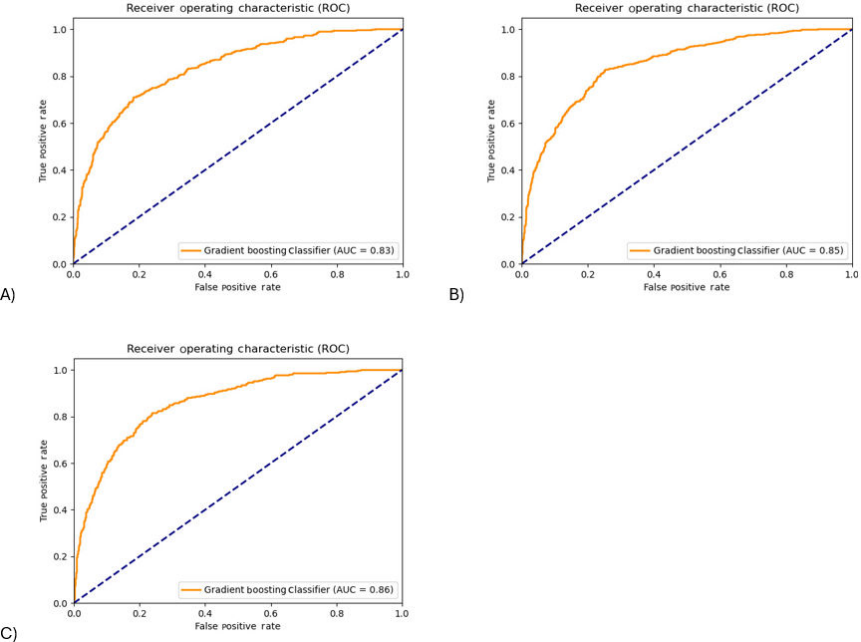
Receiver operating characteristics (ROC) curves of Gradient boosting classifier (identified in the internal validation) in predicting infants at risk of rapid weight gain by the age of 1 year in the testing dataset (external validation). (**A**) Model 1 included maternal prepregnancy BMI, smoking during pregnancy, gestational age, parity, infant sex, and birth weight as predictors. (**B**) Model 2 included model 1 predictors plus any breastfeeding (yes or no) at age 6 months. (**C**) Model 3 included model 2 predictors plus solids introduction (yes or no) at age 6 months. AUC: area under the curve.

**Figure 4.**
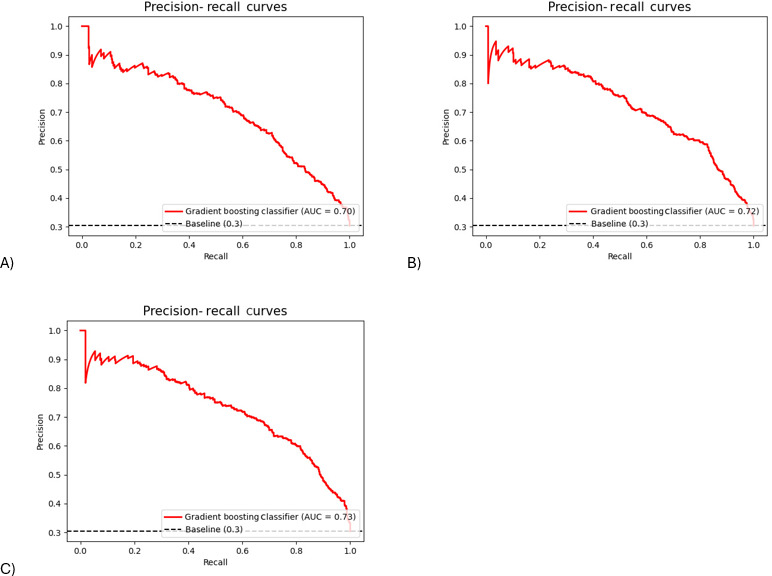
Precision recall curves (PRC) of Gradient Boosting classifier (identified in the internal validation) in predicting infants at risk of rapid weight gain by the age of 1 year in the testing dataset (external validation). (**A**) Model 1 included maternal prepregnancy BMI, smoking during pregnancy, gestational age, parity, infant sex, and birth weight as predictors. (**B**) Model 2 included model 1 predictors plus any breastfeeding (yes or no) at age 6 months. (**C**) Model 3 included model 2 predictors plus solids introduction (yes or no) at age 6 months.

The Gradient Boosting classifier was used to predict infant RWG in the testing dataset. The ability of the Gradient Boosting classifier to distinguish between infants with or without RWG was excellent in the testing dataset with ROC AUC of 0.83‐0.86 for 3 models, respectively (Figure 3). The AUPRC of 3 models spanned from 0.70 to 0.73, indicating a decent model performance (Figure 4). All 3 models (model 1, 2, 3) performed well in terms of accuracy (0.75, 0.78, and 0.78), sensitivity (0.75, 0.78, and 0.80) and specificity (0.76, 0.77, and 0.77), but performed modestly for precision (0.57, 0.60, and 0.60), *F*_1_-score (0.65, 0.68, and 0.69), and Cohen κ (0.47, 0.51, and 0.52). Consistent with the results of the internal validation, model 2 with the addition of breastfeeding improved the predictive performance of model 1 by 0.01-0.04. However, the performance of models 2 and 3 was similar.

### Additional Analyses

The SHAP analyses revealed that birth weight was the most influential factor underlying the prediction of infant RWG, followed by breastfeeding, gestational age, and maternal prepregnancy BMI. In contrast, parity, infant sex, timing of solids introduction, and maternal smoking had relatively less impact on the prediction (Supplementary Figure 3 in Multimedia Appendix 1).

## Discussion

### Principal Findings

Harnessing high-quality prenatal and early postnatal data from 7 Australian and New Zealand cohorts, we developed the first ML models to predict the risk of infant RWG by the age of 1 year (a proxy marker for later obesity risk) using only measures routinely or feasibly collected in primary health care. Our models enable risk prediction in infancy from birth to age 6 months, a critical window to intervene before the development of modifiable obesity-contributing behaviors such as unhealthy dietary intake and low physical activity. The resulting models demonstrated acceptable performance in predicting the risk of infant RWG.

Given the strong association between infant RWG and elevated obesity risk [12-15], early identification of infants at risk of RWG is a crucial step toward early obesity prevention. While research has assessed factors associated with infant RWG [30,41], here we present the first risk prediction models to predict RWG by the age of 1 year. The Gradient Boosting classifier exhibited excellent discrimination (>80%) to distinguish between infants with or without RWG, and performed well in making accurate predictions, particularly true positive cases (with accuracy and sensitivity all exceeding 75% confidence). However, the performance of the model in minimizing false positives was modest as indicated by accuracy, *F*_1_-score, AUPRC, and Cohen κ. Notably, the addition of breastfeeding at age 6 months in risk prediction (model 2) improved model predictive accuracy by 3%‐4% across all performance metrics. However, the inclusion of solids introduction showed minimal impact on model performance. This suggests that acceptable risk prediction can be obtained at birth with very few routinely collected prenatal and birth variables, but the addition of postnatal breastfeeding would further enhance the risk prediction.

### Comparison With Previous Work

Several statistically based risk prediction models have been developed to identify early obesity risk at ages 2‐5 years using predictors collected from birth to age 2 years among children from the United Kingdom [42-45], the United States [46], Israel [47], and New Zealand [48]. Furthermore, various ML-based early childhood obesity risk prediction models have also been developed [49-51]. However, the clinical utility of these models is questionable. For example, the statistical-based models were often derived from a small or a single dataset with external validation being conducted in only 1 or 2 datasets [42-44,48], both of which limit the model predictive accuracy and generalizability. For previous ML-based childhood obesity risk prediction models [49-51], although large eHealth data were used, these models are not clinically feasible as they require over a hundred predictors for risk prediction, undermining utility in primary health care settings.

Studies to evaluate the real-world impact of risk prediction models are the essential next step in the translation pathways [33]. There are, however, important economic, ethical, and clinical challenges for impact evaluation of existing obesity risk prediction models [7]. We proposed a novel and pragmatic solution by using infant RWG as a more acceptable and implementable alternative marker for obesity risk. Apart from systematic reviews [14,15], childhood obesity risk prediction models also consistently found that infant RWG is the most influential predictor of obesity outcomes [43,44,46], providing further evidence to support infant RWG as a valid infancy marker of future obesity risk. Our previous analysis in the current 7 cohorts showed that infant RWG was associated with 4.5 times increased risk of child overweight or obesity by the age of 5 years [31]. This translates to a population attributable fraction of 48.6%, suggesting that about half of child overweight or obesity were attributed to infant RWG with the prevalence of 27%.

Predicting the risk of RWG in infancy offers a pragmatic and low-cost solution for conducting ethically acceptable impact studies to assess clinical impact, as child growth indicators are routinely collected. Our models can be feasibly integrated into routine growth monitoring. Clinicians providing infant care are familiar with the routine use of risk prediction instruments. For example, the neonatal sepsis calculator has been successfully implemented around the world to improve antibiotic stewardship over the past decade [52]. Assessing the risk of infant RWG will raise the awareness of both primary health care professionals and parents about the vital role of early growth in shaping later child health. Research has shown that primary health professionals require support to monitor growth effectively despite growth monitoring being part of the routine infant care [53]. Moreover, both health professionals and parents have poor awareness on the concept and adverse health impact of infant RWG [10,54]. Embedding risk assessment of RWG in routine growth monitoring may motivate families with infants to make behavioral changes and establish positive health behaviors from the beginning of life.

Past studies primarily focused on exploring factors associated with childhood obesity but not infant RWG. Our study is novel in leveraging SHAP analyses to understand the relative contribution of prenatal and postnatal factors in infant RWG prediction. Our finding that birth weight was the most influential factor contributing to infant RWG prediction is supported by postnatal catch-up growth in body weight commonly observed in low-birth-weight infants [55]. It is well known that birth weight is influenced by a range of maternal factors from preconception to pregnancy such as maternal prepregnancy BMI, gestational weight gain, maternal health status, maternal nutrition, and other lifestyle behaviors during pregnancy [56]. Furthermore, our analyses showed that maternal prepregnancy BMI, gestational age, and breastfeeding also played a crucial role in infant RWG prediction, which further highlights the importance of targeting women during preconception and pregnancy to prevent infant RWG.

Leveraging statistical findings, ML, and high-quality cohort data for risk prediction model development is a major strength of the current study, ensuring predictive accuracy, generalisability, and clinical interpretability of the resulting risk prediction models. Statistics focuses on inference, whereas ML focuses on making accurate predictions, it has been increasingly advocated that statistics and ML could be combined to build reliable and clinically useful risk prediction models [57]. The use of statistical inferences to guide our predictor selection improves the clinical interpretability and utility of ML based risk prediction models. On the other hand, ML enables accurate predictions by discovering nuanced relationships between variables and data dimensions beyond “linear” relationship that is often assumed in statistical prediction. Another strength of our study was the use of diverse high-quality data from 7 cohorts, which enhances the model generalisability. We also considered clinical integration during model development by purposely selecting predictor variables readily collectable in primary health care, promoting future clinical integration. Moreover, ML-based risk prediction models can be easily transformed into digital tools that allow automatic risk prediction and convenient integration with electronic management systems in primary health care. Self-assessment by families with newborns or infants is also possible.

### Limitations

Notwithstanding, the limitations of our study should be noted. Some predictors had high levels of missing data. Although missing data imputation was conducted, further model validation with high quality data will be desirable to further enhance the predictive accuracy, generalisability, and clinical utility of our models. Moreover, further research incorporating eHealth records or clinical data for model calibration is needed before clinical integration. However, notably, the use of high-quality cohort data also has its benefit in building accurate models. Our models will serve as a foundation for future validation and calibration with more robust cohort and clinical data. The prevalence of infant RWG across 7 cohorts is comparable to those reported in previous research [14,15]. However, the utility of the resulting model in countries other than Australia and New Zealand requires further investigation. Finally, our model did not capture other genetic or environmental predictors of infant RWG, but it is important to note that we aim to build simple and practical models for clinical integration instead of complex models with superior predictive accuracy but limited clinical utility.

### Conclusions

We developed the first set of ML-based risk prediction models to identify infant RWG, a potent proxy marker denoting obesity risk later in life. The resulting models using routinely collected prenatal and postnatal factors showed good predictive accuracy as evidenced by various predictive performance metrics with gradient boosting classifiers exhibiting the best performance. Our next step is to further calibrate the resulting models using routine clinical data and convert our ML models into a digital tool for use in primary health care or enable self-assessment for families with newborn or infants. Future clinical trials will be conducted to evaluate the real-world utility of our models in identifying RWG and assess the impact of our models along with early interventions in prevention of infant RWG and, in turn, long-term overweight, and obesity.

## Supplementary material

10.2196/69220 Multimedia Appendix 1 Additional tables and figures.

## Abbreviations

AUC: area under the receiver operating characteristic curve
AUPRC: area under the precision-recall curve
BIS: Barwon Infant Study
InFANT: Infant Feeding Activity and Nutrition Trial
ML: machine learning
POI: Prevention of Overweight in Infancy
PRC: precision-recall curve
ROC: receiver operating characteristic
RWG: rapid weight gain
SHAP: Shapley Additive Explanations
SMOTE: Synthetic Minority Over-sampling Technique
WHO: World Health Organization
