# Supplementary material for: Development of Machine Learning–Based Risk Prediction Models to Predict Rapid Weight Gain in Infants: Analysis of Seven Cohorts
Source: JMIR Public Health Surveill. 2025 Jun 18;11:e69220. doi: 10.2196/69220 (PMC12192193; doi:10.2196/69220)
Supplement: Multimedia Appendix 1 [file publichealth-v11-e69220-s001.docx]

**Supplementary materials**

**
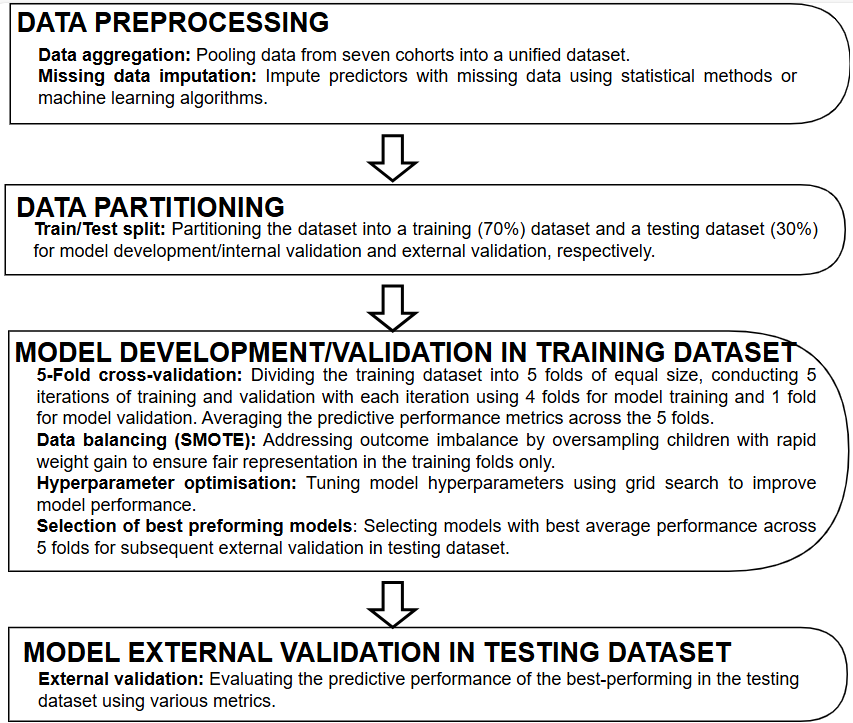
**

**Supplementary Figure 1.** Flow chart showing the development of the machine learning risk prediction models to identify risk of rapid weight gain by age one year.


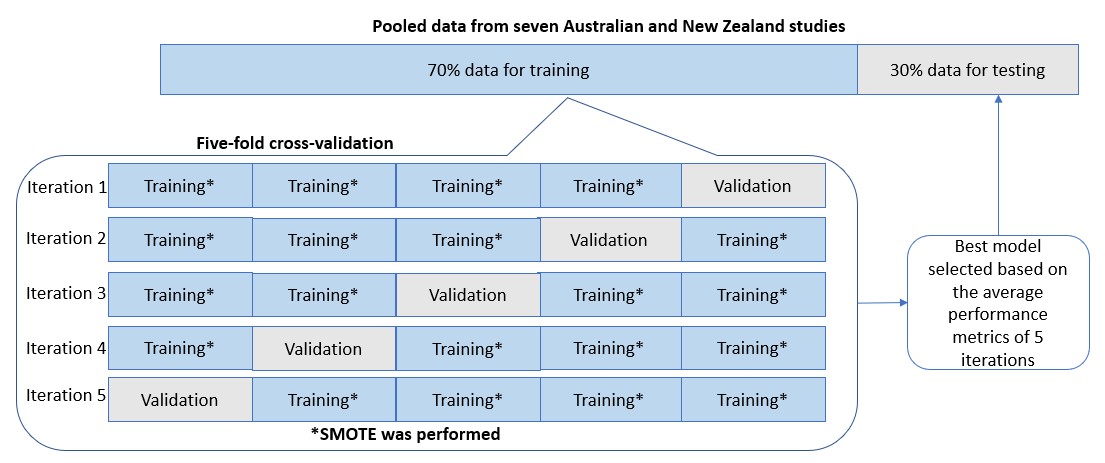


**Supplementary Figure 2**. Detailed model development and validation process including SMOTE to account for imbalanced outcome data

***Missing data imputation***

Maternal smoking during pregnancy (yes vs no) was imputed by mode imputation, whereby missing values were replaced with the most frequent category observed in the dataset. Median imputation was used to impute missing data of maternal pre-pregnancy BMI and gestational age. For breastfeeding and solids introduction, we adopted ML algorithms to impute missing values using infant sex, birth weight and parity as predictors and those with complete data as the training dataset for missing data prediction. AdaBoost classifier was chosen to predict missing values of infant feeding variables as it outperformed other ML algorithms.

**Supplementary Table 1.** Missing data imputation for predictors to predict risk of rapid weight gain by age one year.

|  | n | % missing | Missing data imputation approach |
| --- | --- | --- | --- |
| Maternal pre-pregnancy BMI (kg/m^2^) | 5065 | 3% | Median imputation |
| Maternal smoking during pregnancy (smoker vs non-smoker) | 4284 | 18% | Mode imputation |
| Gestational age (weeks) | 4185 | 20% | Median imputation |
| Child sex (boy vs girl) | 5233 | 0% | No imputation |
| Birth weight (kg) | 5233 | 0% | No imputation |
| Parity (with vs without sibling) | 5233 | 0% | No imputation |
| Any breastfeeding duration at 6 months (yes vs no) | 4879 | 7% | AdaBoost classifier |
| Timing of solids introduction at 6 months (yes vs no) | 3274 | 37% | AdaBoost classifier |

***ML model hyperparameters optimization***

This study employed seven commonly used machine learning (ML) models that are well-suited for similar risk prediction tasks. The hyperparameters of each model play a critical role in controlling the training process, and their optimization is essential for achieving optimal model performance on our specific risk prediction tasks.

To optimize these hyperparameters, we utilized a grid search cross-validation approach. This method systematically explores a range of hyperparameter values, combining them in various ways to determine the best-performing configuration for each model. In total, our script trained 8,843 models with different hyperparameter combinations to identify the most suitable settings for each ML model used in our rapid growth prediction tasks, based on the prediction accuracy. The final optimized hyperparameters for each model are as follows:

**Supplementary Table 2**. Optimised hyperparameters for eight ML algorithms being utlised to predict rapid weight gain by age one year.

| ML Models | Hyperparameters | Ranges |
| --- | --- | --- |
| Logistic Regression | max_iter | [100, 200, 300] |
| Decision Tree Classifier | criterion | ['gini', 'entropy'] |
|  | splitter | ['best', 'random'] |
|  | max_depth | [None, 10, 20, 30, 40, 50] |
|  | min_samples_split | [2, 5, 10] |
|  | min_samples_leaf | [1, 2, 4] |
|  | max_features | [None, 'sqrt', 'log2'] |
| Random Forest Classifier | n_estimators | [100, 200, 300] |
|  | criterion | ['gini', 'entropy'] |
|  | max_depth | [None, 10, 20, 30, 40, 50] |
|  | min_samples_split | [2, 5, 10] |
|  | min_samples_leaf | [1, 2, 4] |
|  | max_features | ['sqrt', 'log2', None] |
|  | bootstrap | [True, False] |
| AdaBoost Classifier | n_estimators | [50, 100, 200] |
|  | learning_rate | [0.01, 0.1, 0.5, 1.0] |
|  | algorithm | ['SAMME', 'SAMME.R'] |
| Gradient Boosting Classifier | n_estimators | [100, 200, 300] |
|  | learning_rate | [0.01, 0.1, 0.05, 0.001] |
|  | max_depth | [3, 4, 5, 6] |
|  | min_samples_split | [2, 5, 10] |
|  | min_samples_leaf | [1, 2, 4] |
|  | subsample | [0.8, 0.9, 1.0] |
|  | max_features | ['sqrt', 'log2'] |
| Support Vector Classifier | C | [0.1, 1, 10, 100] |
|  | kernel | ['linear', 'poly', 'rbf', 'sigmoid'] |
|  | gamma | ['scale', 'auto'] |
|  | degree | [2, 3, 4] |
|  | probability | [False, True] |
| K-Neighbours Classifier | n_neighbors | [3, 5, 7, 9, 11] |
|  | weights | ['uniform', 'distance'] |
|  | algorithm | ['auto', 'ball_tree', 'kd_tree', 'brute'] |
|  | p | [1, 2] |
| Multi-Layer Perceptron (MLP) Classifier | hidden_layer_sizes | [(100,), (100, 50), (100, 100, 50)] |
|  | activation | ['identity', 'logistic', 'tanh', 'relu'] |
|  | solver | ['sgd', 'adam'] |
|  | alpha | [0.0001, 0.001, 0.01] |
|  | learning_rate | ['constant', 'invscaling', 'adaptive'] |
|  | learning_rate_init | [0.001, 0.01, 0.1] |
|  | max_iter | [200, 400, 600] |
|  | early_stopping | [True, False] |

**Supplementary Table 3**. Predictive performance metrics used to evaluate the risk prediction models to identify risk of rapid weight gain (RWG) by age one year.

| **Predictive performance metrics** | **Definition/ Calculation** | **Interpretation** |
| --- | --- | --- |
| Area Under the ROC Curve (AUC) | AUC is the area under the Receiver Operating Characteristic (ROC) curve  The ROC curve is a plot of the true positive rate (x-axis) against the false positive rate (y-axis) across various classification threshold | Ability of the model to distinguish between positive and negative cases  The closer the curve towards the left hand of the y-axis, the better the model prediction. |
| Accuracy | Proportion of correct predictions (both true positives and true negatives)  The number of correct predictions (both true positives and true negatives) divided by total number of predictions | Overall correctness of the model |
| Precision  (positive predictive value) | Proportion of true positive instances out of all instances predicted as positive by the model.  True positives divided by the sum of true and false positives | Model's ability to predict true positives, while minimising false positives |
| Sensitivity  (recall, true positive rate) | Proportion of true positives identified by the model.  True positives divided by the sum of true positives and false negatives | Model's ability to correctly classify all true positives |
| Precision Recall Curve (PRC) | The PRC plots recall (y-axis) against precision (x-axis) across different thresholds | The closer the curve towards the right hand of the y-axis, the better the model prediction. |
| F1-score | (The product of precision and sensitivity divided by the sum of precision and sensitivity) times 2 | Balanced performance of the model that accounting for both precision and sensitivity |
| Specificity  (true negative rate) | Proportion of true negatives correctly identified by the model  True negatives divided by the sum of true negatives and false positives | Model's ability to correctly identify true negatives |
| Cohen’s Kappa | Agreement between predicted and observed outcome accounting for agreement by chance  (Observed agreement minus expected agreement) divided by (1 minus expected agreement) | Higher values indicate better agreement between the model and actual outcomes |

True positives: Infants identified as having RWG when they have RWG

True negatives: Infants identified as have no RWG when they have no RWG

False positives: Infants incorrectly identified as having RWG when they do not have RWG

False negatives: Infants incorrectly identified as have no RWG by the model, but have RWG


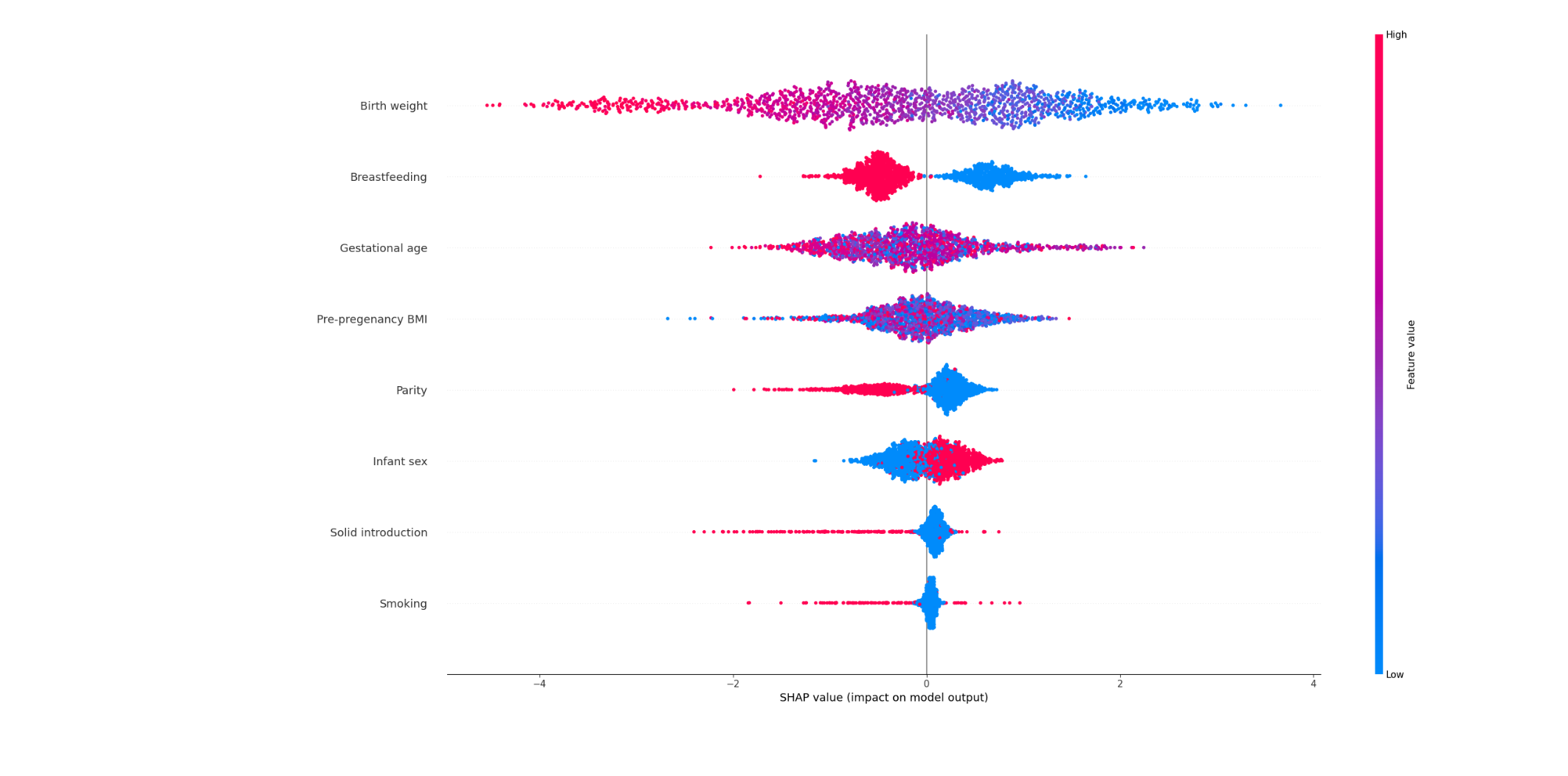


**Supplementary Figure 3.** The SHapley Additive exPlanations (SHAP) beeswarm plot illustrating the contribution of prenatal and postnatal factors in the model prediction of infant rapid weight gain (RWG). The horizontal axis displays SHAP values, which quantify how much each feature shifts the prediction relative to a baseline. Points to the right indicate an increased likelihood of infant RWG, while points to the left suggest a decreased likelihood of infant RWG. The vertical axis lists the features in order of importance, with those at the top (e.g., birth weight, breastfeeding, gestational age, and maternal pre-pregnancy BMI) having the most substantial impact. Each dot represents an individual case, and its colour reflects the actual feature value (red for higher values and blue for lower ones), which helps illustrate trends, such as higher birth weight typically pushing predictions upward. Additionally, the spread of dots for each feature shows the variability in their impact across different cases, providing a nuanced view of how each factor influences the model’s overall prediction.
